# Survival of 48866 cancer patients: results from Nantong area, China

**DOI:** 10.3389/fonc.2023.1244545

**Published:** 2023-08-11

**Authors:** Gao-Ren Wang, Hong Xu, Hai-Zhen Chen, Yong-Sheng Chen, Zhuo-Jian Ni, Li-Yun Fan, Ai-Hong Zhang, Pei-Pei Xu, Yun Qian, Bo Cai, Jian-Guo Chen

**Affiliations:** ^1^ Department of Epidemiology, Nantong Tumor Hospital, Affiliated Tumor Hospital of Nantong University, Nantong, China; ^2^ Department of Chronic Disease Prevention and Control, Nantong Center for Disease Control and Prevention, Nantong, China; ^3^ Department of Epidemiology, Qidong Liver Cancer Institute, Qidong People’s Hospital, Affiliated Qidong Hospital of Nantong University, Qidong, China; ^4^ Department of Chronic Disease Prevention and Control, Haimen Center for Disease Control and Prevention, Haimen, China; ^5^ Department of Chronic Disease Prevention and Control, Tongzhou Center for Disease Control and Prevention, Tongzhou, China; ^6^ Department of Chronic Disease Prevention and Control, Rudong Center for Disease Control and Prevention, Rudong, China; ^7^ Department of Chronic Disease Prevention and Control, Rugao Center for Disease Control and Prevention, Rugao, China; ^8^ Department of Chronic Disease Prevention and Control, Hai’an Center for Disease Control and Prevention, Hai’an, China

**Keywords:** neoplasm, survival, follow-up, rural area, hospital-based cancer registry, population-based cancer registry

## Abstract

**Objective:**

This study aimed to provide a realistic observation of survival by major site for 48,866 cancer patients treated at a tertiary cancer hospital in a rural area of China.

**Methods:**

Patients with cancer registered between 2007 and 2017 in the Nantong rural area were followed up. The starting date for survival calculation was the date of the first diagnosis of cancer at the Nantong Tumor Hospital, and the closing date was December 31, 2020. Observed survival (OS) was analyzed according to ICD-10 site, sex, age, region, and hospitalization period using the life table method and compared using the Wilcoxon (Gehan) statistic.

**Results:**

The overall 5-year OS rate was 40.48% for all 48,866 patients, 30.19% for males, and 51.90% for females. The top five cancer sites, accounting for 60.51% of the total cases, were the esophagus, lung, stomach, liver, and cervix, with 5-year OS rates of 33.72%, 18.64%, 32.10%, 19.04%, and 71.51%, respectively. The highest 5-year OS was observed in the thyroid (87.52%) and the lowest was in the pancreas (6.37%). Survival was significantly higher in younger patients than in older patients, with 5-year OSs of 69.26% and 19.84% in those aged 20-29 and 90-99 years, respectively. Five-year OSs improved significantly from 39.35% in 2007-2011 to 41.26% in 2012-2017.

**Conclusion:**

Overall survival improved over the years, although the improvement at some sites was not significant. The observed survival varies from region to region, reflecting differences in the patterns of major sites, disparities in proportions of hospitalization, and demographic characteristics.

## 1 Introduction

Monitoring the survival of patients with malignancies (cancers) is an important component of cancer management. Currently, studies evaluating cancer survival worldwide, including EUROCARE, CONCORD, CANSURV, and others (1–3), have played a significant role in the global evidence base for cancer control and international comparison of the effectiveness of healthcare systems (4). In the “Healthy China Action plan” (2019-2030) released in 2019, the Chinese government included cancer survival as an essential indicator in assessing the level of care provided for patients with cancer, and aimed to raise the cancer survival from 40.5% in 2015 (5) to 43.3% in 2022, and 46.6% in 2030 (6). Currently, there is limited research on cancer survival in China. Population-based cancer registries (PBR) cover only 477 million people, less than one-third of the national population, and of these, only 27.60% have valid registration data (7, 8). In addition, hospital-based cancer registries (HBR) are poorly functioning, and reports on cancer survival are limited to a few cancer registries in China (9, 10) and other countries (11, 12), or mostly, only focus on cancer survival in site-specific cancers (13–21).

The Nantong Tumor Hospital initiated an HBR in 2002 with the aim of descriptively analyzing the clinical distribution characteristics of cancer patients hospitalized at our institution and to carry out follow-up studies. After 2013, multiple follow-up visits (including home visits) were conducted to determine survival outcomes, and survival studies were conducted in a subset of patients from the database (9). In this study, we analyzed the survival outcomes of all cancer patients who were registered at our institution between 2007 and 2017 in the rural area of Nantong, China.

## 2 Materials and methods

### 2.1 Regional background

Nantong City is located in the eastern part of Jiangsu Province and on the northern bank of the Yangtze River, facing Shanghai and Suzhou in Jiangsu Province across the river. At the end of 2021, it had a population of 7.73 million, with an area of 8,001 Km^2^, including three urban areas and six counties (including two county-level cities, Tongzhou and Haimen) that were incorporated into the Nantong urban district in 2009 and 2020, respectively. According to the China’s urban and rural division standards and traditional jurisdiction, the rural areas referred to in this paper are based on the division of the 6 counties (or county-level cities) in 2007 (22) (hereinafter referred to as the “rural area” or “6 regions”).

### 2.2 Cancer registration

China launched the “Cancer Registration and Follow-up Project” nationwide since 2008 and issued the “Cancer Registration Management Measures” (23), which includes cancer follow-up in 2015. Nantong City has established PBRs since 2010 in all counties and districts in accordance with the requirements of China’s National Cancer Registration Center, and the registration data on cancer after 2011 were included in the China Cancer Registration Annual Report (24). Before this, the Qidong Cancer Registry was established as early as 1972, and the cancer registration work in Haimen and Hai’an started in 1999 and the beginning of the 21st century, respectively, and the related data of these three PBRs have been adopted and reported in relevant literature (5, 9, 25, 26). The HBR at Nantong Tumor Hospital was established in 2002 based on the hospital’s health information system (HIS) (9, 27).

### 2.3 Sources of patients

All patients were enrolled at the Nantong Tumor Hospital, which is located in the northwestern part of the city. It is the only tertiary cancer hospital in the northern area of Jiangsu province, and its services extend to the northern part of the province as well as to parts of other provinces, such as Anhui and Shandong (28). Data on patients discharged information from 2002 to 2017 were imported from the HIS, excluding patients with non-tumors and benign tumors. A hospital-based cancer registry database was established and refined through multiple steps of data screening and collating, using patient identification information and residence information. Between 2002 and 2017, a total of 302,471 records were registered, of which 251,022 were related to malignant tumors, corresponding to 100,740 patients. Among them, 74,503 patients with malignancies from urban and rural areas of Nantong accounted for 73.96% of all patients hospitalized during this period.

### 2.4 Follow-up methods

Active HBR Follow-up: Based on personal information (ID number, telephone number, and address of family members) registered during the hospitalization of patients, telephone follow-up (active follow-up) was conducted to obtain information on their survival status. Approximately 25% of the patients (or their family members) were contacted, and their outcomes were promptly updated.

Mixed follow-up of PBR: Due to changes or errors in the telephone numbers or addresses of hospitalized patients, and where patients who were unreachable (e.g., being out of their home or not answering the phone), multiple rounds of follow-up were conducted through PBRs in the Nantong area in 2013, 2020, and 2021. These follow-up efforts included passive data retrieval and verification with each PBR, as well as on-site active follow-up based on the patient information provided by the HBRs. The primary field used to match hospital registration cases with population registration cases was the patient’s identification number. In cases where individual identification numbers could not be matched due to incorrect or missing ID information, then name, address, or other alternative fields (variables) were used for matching purposed. For individuals identified as “survivors” in both the HBR and PBR databases, an additional phase of on-site “active follow-up” or “home visit” was conducted by local health workers in the Prevention Network to determine the patients’ survival outcomes.

**Figure 1:**
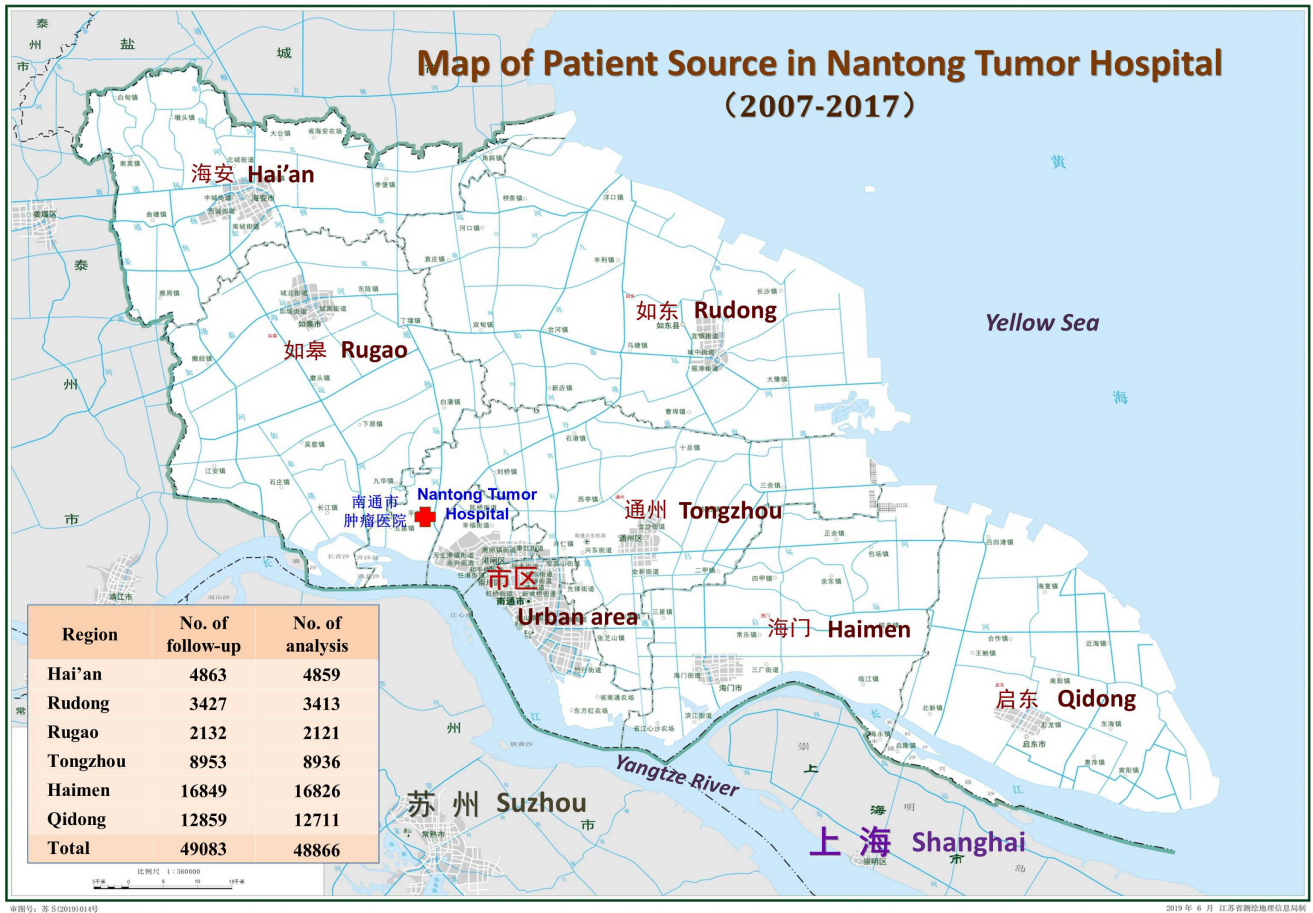
Distribution of cancer patients from rural area in Nantong.

Because three out of the six counties in this area had unified cancer registration after 2010, to ensure the reliability of patient follow-up data, this data adopted the follow-up outcome information recorded by the HBR from 2007 (i.e., pooled data of HBR cases pushed forward by the PBR for 3 years). From 2007 to 2017, a total of 57,922 patients needed to be followed up; of them 8839 cases in the “old” urban districts of Nantong city were ruled out, leaving 49,083 cases belong to “rural” cases. Furthermore, 217 cases of non-local household registered residents, emigration, and non-malignancies were excluded; hence, 46,688 cases were eventually included in the analysis, accounting for 99.56% (**Figure 1**).

### 2.5 Statistical methods

*Date of cancer diagnosis:* Cancer patients are usually admitted to a hospital repeatedly. The starting date for survival calculation in this study was the date of the first diagnosis of cancer (hospitalization) at the Nantong Tumor Hospital.

*Closing date of follow-up:* The closing date: December 31, 2020. If a patient died before that date, the deadline was the patient’s actual date of death. The actual date of death for some patients occurred during the 2021 follow-up period, but in our data processing, the patient was still defined as a “survivor,” which is known as a “right censoring” case.

*Variable settings:* The variables involved in this analysis included ICD tumor site (by system category), sex (male, female), age (0-14, 15-34, 35-59, 60-79, 80-99 [80 and above]), place of household registration (Haian: HA, Haimen: HM, Qidong: QD, Rudong: RD, Rugao: RG, and Tongzhou: TZ), and the period (2007-2011, 2012-2017), etc.

*Statistical processing:* The Data were analyzed using IBM SPSS Statistics version 22. The observed survival (OS) and the standard errors (SE) by ICD-10 site and by region, sex, age group, and period were calculated using the actuarial (life-table) method and compared using the Wilcoxon (Gehan) statistic. Statistical P was set than 0.05.

## 3 Results

### 3.1 General characteristics of cases

*Annual hospitalizations for malignancies:* The number of hospitalized cases from this rural area was approximately 3,600 to 3,700 per year during the study period (2007-2010), and increased to 5,300 to 5,800 per year in the last three years (2015-2017), indicating a rising trend in the number of cases. Among 48,866 patients included in the analysis, 25,728 (52.65%) were men and 23,138 (47.35%) were women, with a sex ratio of 1.11:1. In malignancies of non-sex-specific sites, the highest ratio of males to females was observed in laryngeal cancer (23.67:1), followed by bladder cancer (4.05:1), liver cancer (3.66:1), nasopharyngeal cancer (2.53:1), gastric cancer (2.47:1), and lung cancer (2.13:1). The lowest sex ratio was observed in breast cancer (0.01:1), followed by thyroid cancer, 0.40:1 (**Supplementary S1**).

*Distribution of cancer patients by region and by age group:* Among the six regions in the area, Rugao (RG) had the largest number of hospitalized patients (16,826), followed by Tongzhou (TZ:12,711) and Rudong (RD:8,936); Qidong (QD) had the lowest number of patients (2,121), followed by Haimen (HM:3,413) and Hai’an (HA:4,859) (**Figure 1**). The top five cancer sites were the esophagus (8718 cases), lungs (7824), stomach (4760), cervix (4449), and liver (3820), accounting for 60.51% (29571/48866) of the total cases. The next most common sites were the breast (3291), rectum (1819), non-Hodgkin lymphoma (1356), colon (1289), and ovary (1152), accounting for 18.23% (8907/48866). Thus the top ten cancers accounted for 78.74% of the total. The highest number of cases occurred between 60-79 years of age (53.81%), followed by 35-59 years (39.29%), 80-99 years (4.99%), 15-35 years (1.82%), and 0-14 years (0.10%) (**Supplementary S2**).

### 3.2 Overall observed survival of malignancies

The 1-, 3-, 5-, 8-, 10-, and 12-year observed survival (OS) for all malignancy sites in this series were 69.38%, 47.49%, 40.48%, 35.07%, 32.42%, and 30.0%, respectively. The sites with the highest 5-year OSs were the thyroid (87.52%), corpus uteri (77.71%), penis (73.13%), breast (73.05%), and cervical uteri (71.51%). The sites with the lowest 5-year OSs were the pancreas (6.37%), gallbladder (10.75%), leukemia (14.2%), lung (18.64%), and liver (19.04%) as shown in **Table 1**.

**Table 1:** Observed survival and standard error (%) by site.

ICD-10	1-Yr		3-Yr		5-Yr		8-Yr		10-Yr		12-Yr	
	OS	SE	OS	SE	OS	SE	OS	SE	OS	SE	OS	SE
**C11**	86.81	1.22	68.67	1.68	59.99	1.81	48.27	2.04	45.18	2.13	39.38	2.61
**C00-14**	74.92	1.45	54.11	1.67	45.90	1.70	39.82	1.78	37.29	1.88	34.05	2.14
**C15**	69.53	0.49	41.97	0.53	33.72	0.51	27.17	0.51	23.98	0.53	20.89	0.57
**C16**	63.09	0.70	38.47	0.71	32.10	0.69	27.99	0.69	24.99	0.72	23.03	0.78
**C17**	67.11	3.81	45.39	4.04	37.72	4.03	28.59	4.18	24.90	4.38	24.90	4.38
**C18**	72.77	1.24	54.93	1.39	48.33	1.42	44.52	1.51	39.30	1.80	37.75	1.94
**C19-21**	83.40	0.87	61.79	1.14	52.89	1.20	46.32	1.29	43.24	1.40	39.52	1.65
**C22**	45.52	0.81	25.99	0.71	19.04	0.65	14.49	0.63	12.56	0.65	10.83	0.74
**C23-24**	36.14	2.26	15.74	1.71	10.75	1.50	8.75	1.43	7.95	1.50	5.96	2.06
**C25**	22.66	1.41	8.68	0.95	6.37	0.82	5.81	0.80	4.89	0.79	4.10	0.85
**C26**	41.18	11.94	23.53	10.29	23.53	10.29	23.53	10.29	23.53	10.29	–	–
**C30-31**	67.97	3.77	49.67	4.04	40.52	4.02	32.52	4.11	29.82	4.20	26.98	4.66
**C32**	77.48	2.80	62.16	3.25	51.60	3.46	47.63	3.63	41.40	4.10	41.40	4.10
**C33-34**	55.02	0.56	25.56	0.49	18.64	0.45	13.99	0.46	12.81	0.48	10.68	0.55
**C37-38**	74.17	3.56	56.29	4.04	45.49	4.12	40.07	4.46	37.96	4.70	34.66	5.32
**C40-41**	63.54	4.91	37.50	4.94	30.44	4.79	23.47	4.82	21.00	4.90	21.00	4.90
**C43**	75.55	2.84	48.91	3.30	36.44	3.25	31.61	3.41	26.15	3.77	26.15	3.77
**C44**	89.06	1.46	70.90	2.12	60.77	2.34	46.23	2.74	40.18	2.98	35.25	3.35
**C45-49**	74.01	3.30	55.93	3.73	46.98	3.79	42.48	3.85	40.19	3.97	32.65	5.08
**C50**	91.58	0.48	78.97	0.71	73.05	0.79	66.19	0.92	63.00	1.03	60.99	1.15
**C51-58**	87.05	1.84	64.76	2.62	57.28	2.77	52.13	3.02	48.75	3.27	47.48	3.42
**C53**	91.82	0.41	76.71	0.63	71.51	0.68	66.54	0.76	63.83	0.82	61.01	0.95
**C54**	93.07	0.89	82.38	1.33	77.71	1.48	72.68	1.74	70.00	1.99	68.30	2.29
**C56**	79.86	1.18	54.69	1.47	43.14	1.49	34.75	1.54	32.45	1.60	31.76	1.71
**C60**	75.29	4.68	60.00	5.31	55.15	5.41	48.87	5.64	48.87	5.64	48.87	5.64
**C61**	78.87	1.87	56.90	2.27	43.27	2.37	31.54	2.86	24.96	3.74	19.97	5.37
**C62-63**	82.22	5.70	77.78	6.20	73.13	6.64	70.32	6.96	64.70	8.37	64.70	8.37
**C64-68**	71.09	2.33	56.50	2.55	51.64	2.59	49.90	2.65	45.94	2.97	45.94	2.97
**C67**	78.14	1.88	62.68	2.20	56.21	2.28	50.73	2.42	47.25	2.59	47.25	2.59
**C69**	80.00	12.65	60.00	15.49	40.00	15.49	40.00	15.49	40.00	15.49	20.00	16.12
**C70-72**	64.22	2.65	42.20	2.73	31.00	2.60	25.67	2.62	23.65	2.79	21.83	3.11
**C73**	94.38	0.85	89.44	1.14	87.52	1.24	81.49	1.89	77.09	2.51	73.84	3.03
**C74-75**	58.33	10.06	33.33	9.62	33.33	9.62	33.33	9.62	33.33	9.62	33.33	9.62
**C76-80**	62.41	1.22	45.21	1.25	40.41	1.24	37.21	1.24	35.59	1.25	33.87	1.26
**C81**	71.88	4.59	52.08	5.10	46.68	5.11	44.05	5.15	44.05	5.15	44.05	5.15
**C82-85**	67.48	1.27	50.07	1.36	43.03	1.37	37.85	1.41	35.55	1.48	32.71	1.68
**C88-90**	73.40	4.56	37.23	4.99	26.50	4.68	22.26	4.83	17.81	5.55	8.90	6.88
**C91-96**	45.71	5.95	18.57	4.65	14.22	4.19	12.55	4.01	12.55	4.01	12.55	4.01
**C97**	73.82	3.18	46.60	3.61	33.52	3.63	20.54	4.60	10.15	4.90	5.08	4.35
**C00-97**	69.28	0.21	47.49	0.23	40.48	0.23	35.07	0.23	32.42	0.25	30.00	0.27

ICD-10, International classification for diseases - 10th version; OS, Observed survival; SE, Standard error. "-", not available.

### 3.3 Survival of malignancies by sex

**Figure 2:**
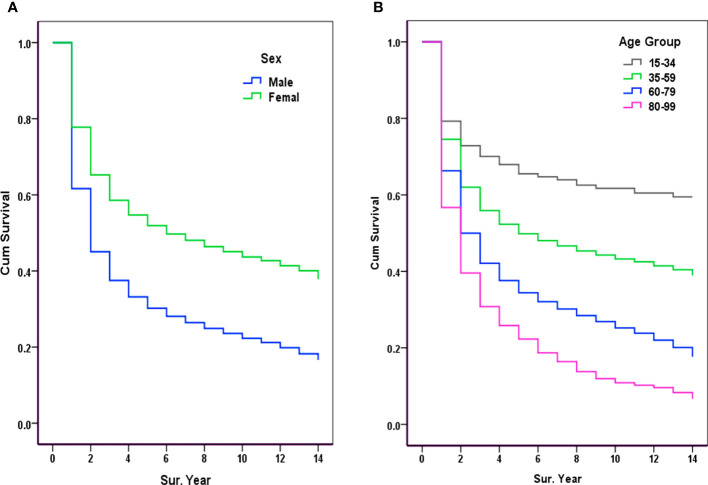
Observed survival of malignancy by sex and age group (2007-2017). **(A)** Sex: Gehan statistic is 2601.58, *P*<0.0000; **(B)** Age group: Gehan statistic is 1493.13, *P*<0.0000.

In this series of malignant cases, survival was significantly lower in men than in women; for males, the 1-, 3-, 5-, 8-, 10-, and 12-year OSs were 61.64%, 37.52%, 30.19%, 24.89%, 22.30%, and 19.81%, respectively; for females, there were 77.78%, 58.57%, 51.90%, 46.37%, 43.67%, and 41.38%, respectively, as shown in **Table 2**. Comparison of OS curves between men and women showed a statistically significant difference; the Gehan statistic was 2601.58, P < 0.0000, as shown in **Figure 2A**.

**Table 2:** Observed survival (%) by site and by sex.

ICD-10	1-Yr		3-Yr		5-Yr		8-Yr		10-Yr		12-Yr	
	Male	Female	Male	Female	Male	Female	Male	Female	Male	Female	Male	Female
C11	85.97	88.94	65.94	75.58	56.92	67.79	43.93	59.50	41.30	55.25	34.71	51.56
C00-14	71.80	80.50	49.21	62.89	41.09	54.50	34.41	49.53	31.54	47.87	28.77	43.50
C15	68.06	72.64	39.66	46.84	31.50	38.39	24.87	32.00	22.04	28.06	19.03	24.77
C16	63.65	61.71	38.09	39.39	31.37	33.89	26.88	30.70	23.61	28.30	21.62	26.41
C17	69.66	63.49	46.07	44.44	37.24	38.32	30.67	24.78	30.67	17.70	30.67	17.70
C18	73.02	72.46	54.80	55.09	46.92	50.00	44.14	45.08	37.36	41.38	35.06	40.43
C19-21	82.53	84.72	59.78	64.86	49.71	57.69	43.08	51.24	40.13	47.95	36.02	44.88
C22	44.43	49.51	24.97	29.76	18.37	21.47	14.11	15.86	12.24	13.71	10.06	13.71
C23-24	35.78	36.48	13.76	17.60	9.01	12.37	6.07	11.10	3.64	11.10	3.64	7.93
C25	22.16	23.32	7.78	9.84	5.17	7.96	4.71	7.29	4.32	5.56	3.81	4.33
C26	45.45	33.33	27.27	16.67	27.27	16.67	27.27	–	27.27	–	–	–
C30-31	67.01	69.64	43.30	60.71	31.95	55.07	27.59	41.80	22.39	41.80	18.32	41.80
C32	77.93	66.67	61.97	66.67	51.55	53.33	47.42	53.33	41.16	–	41.16	–
C33-34	51.17	63.20	22.65	31.76	16.42	23.35	12.34	17.58	11.29	16.10	8.96	15.16
C37-38	69.05	80.60	50.00	64.18	39.70	52.79	31.24	52.79	27.77	52.79	23.14	52.79
C40-41	54.90	73.33	27.45	48.89	24.71	36.94	18.53	28.28	12.35	28.28	12.35	28.28
C43	73.50	77.68	47.86	50.00	34.54	38.42	29.76	33.48	22.37	30.69	22.37	30.69
C44	87.55	90.63	68.24	73.66	55.39	66.51	42.66	50.02	37.07	43.48	31.31	39.46
C45-49	72.29	75.53	50.60	60.64	41.75	51.57	35.95	48.41	31.97	48.41	23.45	42.72
C50	83.87	91.66	74.19	79.02	63.21	73.14	38.77	66.40	19.39	63.37	19.39	61.34
C51-58	–	87.05	–	64.76	–	57.28	–	52.13	–	48.75	–	47.48
C53	–	91.82	–	76.71	–	71.51	–	66.54	–	63.83	–	61.01
C54	–	93.07	–	82.38	–	77.71	–	72.68	–	70.00	–	68.30
C56	–	79.86	–	54.69	–	43.14	–	34.75	–	32.45	–	31.76
C60	75.29	–	60.00	–	55.15	–	48.87	–	48.87	–	48.87	–
C61	78.87	–	56.90	–	43.27	–	31.54	–	24.96	–	19.97	–
C62-63	82.22	–	77.78	–	73.13	–	70.32	–	64.70	–	64.70	–
C64-68	69.01	74.81	55.79	57.78	49.99	54.58	48.66	52.23	43.73	50.01	43.73	50.01
C67	79.95	70.83	63.50	59.38	56.31	55.91	49.67	55.91	45.51	55.91	45.51	55.91
C69	66.67	100.00	33.33	100.00	16.67	75.00	–	75.00	–	75.00	–	37.50
C70-72	57.39	72.19	35.23	50.33	25.51	37.38	22.11	29.66	20.73	26.70	18.75	26.70
C73	90.95	95.76	82.38	92.29	78.38	91.22	73.24	84.87	67.13	81.17	63.40	78.10
C74-75	61.54	54.55	30.77	36.36	30.77	36.36	30.77	36.36	–	36.36	–	36.36
C76-80	56.62	67.86	38.79	51.23	34.24	46.20	31.18	42.85	29.45	41.27	28.09	39.23
C81	74.14	68.42	50.00	55.26	44.39	50.00	42.05	46.97	42.05	46.97	42.05	46.97
C82-85	64.19	71.95	48.21	52.61	41.14	45.62	35.39	41.26	32.12	40.41	29.16	37.73
C88-90	66.67	80.43	31.25	43.48	22.53	30.60	16.27	30.60	10.84	30.60	10.84	0.00
C91-96	41.46	51.72	19.51	17.24	14.45	13.79	11.24	13.79	11.24	13.79	11.24	13.79
C97	71.29	76.67	38.61	55.56	26.18	41.80	18.15	23.45	12.10	–	6.05	–
C00-97	61.64	77.78	37.52	58.57	30.19	51.90	24.89	46.37	22.30	43.67	19.81	41.38

ICD-10, International classification for diseases - 10th version. "-", not available.

### 3.4 Observed survival by age group

Survival rates were significantly higher in younger cancer patients, with 5-year OSs rates of 69.26% and 60.87% in those aged 20-29, and 30-39 years, respectively. Survival declined with increasing age, with 5-year OSs of 22.83% and 19.84% for those aged 80-89 and 90-99 years, respectively. The age-specific OS curves showed a stable gradient and decreasing trend with increasing age and survival, with a Gehan Statistic of 1493.13, P<0.0000, as shown in **Figure 2B**.

### 3.5 Observed survival in major cancer sites

**Figure 3:**
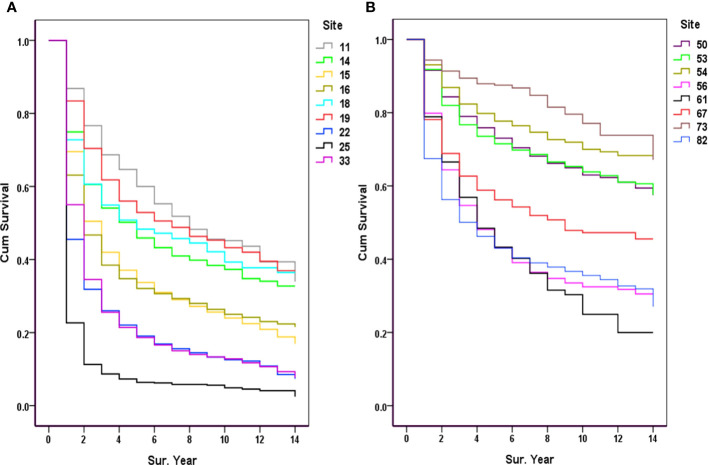
Observed survival of the major cancers (2007-2017). **(A)** 11, Nasopharynx; 14, Lip, oral & pharynx [Except Nasopharynx]; 15, Esophagus; 16, Stomach; 18, Colon; 19, Rectum & anus; 22, Liver; 25, Pancreas; 33, Trachea & lung; **(B)** 50, Breast; 53, Cervix uteri; 54, Corpus uteri; 56, Ovary; 61, Prostate; 67, Bladder; 73, Thyroid gland; 82, Non-Hodgkin’s lymphoma [NHL].

Among the major sites of malignancies sorted by the number of hospitalizations, the lowest 5-year survival rates were for pancreatic cancer (6.37%), lung cancer (18.64%), liver cancer (19.04%), gastric cancer (32.10%), and esophageal cancer (33.72%) (**Figure 3A**); The highest 5-year survival rates were for thyroid cancer (87.52%), corpus cancer (77.71%), breast cancer (73.05%), cervical cancer (71.51%), and bladder cancer (56.21%), as shown in **Figure 3B**.

### 3.6 Survival of cancer sites by period

The 11-year data were subdivided into two periods: 2007-2011 and 2012-2017. The results showed an improvement in the 5-year OSs in the latter period (P<0.01). The OSs were significantly improved for cancers of the oral cavity and lung, and significantly decreased for cancers of the esophagus and cervix, but was not statistically significant for other cancers between the two periods, as shown in **Table 3**.

**Table 3:** Comparison of 5-year observed survival (%) for major cancer sites by period.

ICD-10	2007-2011			2012-2017			Wilcoxon (Gehan) Statistic	Sig.
	Number Entering Interval	OS	SE	Number Entering Interval	OS	SE		
C11	207	58.05	2.72	235	61.57	2.42	3.18	0.0746
C00-14	166	42.17	2.64	229	48.57	2.22	4.96	0.0259^*^
C15	1437	34.80	0.78	1510	32.93	0.68	12.29	0.0005
C16	691	33.02	1.07	765	31.48	0.90	3.02	0.0821
C18	166	46.29	2.72	377	49.17	1.67	0.23	0.6331
C19-21	335	51.64	2.03	533	53.58	1.48	0.98	0.3219
C22	329	16.82	0.93	409	20.90	0.90	3.12	0.0775
C33-34	475	15.39	0.70	905	20.39	0.59	28.16	0.0000
C44	110	62.80	3.77	149	59.57	2.99	0.47	0.4932
C50	847	69.44	1.35	1333	75.19	0.97	3.82	0.0508
C53	1313	73.16	1.06	1663	70.51	0.89	16.08	0.0001
C54	195	76.52	2.70	384	78.31	1.76	0.94	0.3312
C56	209	44.32	2.39	288	42.27	1.92	0.87	0.3515
C67	110	60.34	3.71	153	53.88	2.88	0.07	0.7930
C73	117	78.38	3.38	391	89.94	1.27	1.82	0.1768
C82-85	245	43.52	2.18	309	42.82	1.75	0.004	0.9524
C00-97	7960	39.35	0.36	10746	41.26	0.29	15.06	0.0001

ICD-10, International classification for diseases – 10th version; OS, Observed survival; SE, Standard error.

### 3.7 Observed survival by region

Due to the uneven number of cases across the six regions and the different proportions of major cancer sites, the overall 5-year OSs of malignancies were not consistent. The relatively low 5-year OSs rates for HM and QD cancer cases were 33.45% and 38.02%, respectively. The relatively high 5-year OSs for the HA and TZ groups were 42.33% and 41.83%, respectively.

## 4 Discussion

The survival of cancers in 17 quality-completed population-based cancer registries (PBR) from 2003 to 2005 was first reported in China in 2015, covering approximately 21.6 million people (29). Soon after, the survival of these 17 PBRs from 2003 to 2015 was updated in 2018 (5). These 17 PBRs were located mainly in eastern China (11 in Jiangsu Province), and most of them were “rural” data, including the PBRs in QD and HM in the Nantong area. However, survival data from hospital-based registries (HBR) are rarely reported (10), and there are no available HBR data that represent a region, except for previously reported data on hospitalized patients in QD and HM (9). This analysis extended the data to rural regions throughout the Nantong area.

This study used mixed methods of repeated active and passive follow-up to maximize the reliability and completeness of the regional follow-up by combining HBR and PBR data within the area, which is crucial for assessing cancer prognosis and conducting cancer survival studies. Because of the regional characteristics of the Nantong Tumor Hospital, more than 75% of cases came from local rural areas; hence, the HBR data are highly representative of the survival status of local rural patients. In this study, the survival outcomes of the majority of patients, based on their clear location and throughout the local CDC reporting systems, were obtained by multiple active and passive follow-ups (visits).

A report from China showed that the survival of cancer patients treated at the Shanghai Cancer Hospital was relatively high, with an overall 5-year survival of 71.0% (10), far higher than the reported 40.5% in China from to 2012-2015 (5). In this Shanghai series, the 5-year survival rate of patients with thyroid cancer was 98.8%, breast cancer was 89.2%; and for cancers of the colon, rectum, stomach, lung, esophagus, and liver, it was 64.6%, 54.5%, 49.0%, 43.1%, and 22.3%, respectively. The lowest 5-year survival rate was 11.4% for pancreatic cancer (10). However, the survival rate may be overestimated because of the low follow-up rate (e.g., 74.6% in the Shanghai series) based on the delayed follow-up practice in our country’s cancer registry and the statistical principles of survival analysis. Furthermore, this hospital-based registry performed passive follow-up results from various provinces and cities (CDCs), the patient source was not described, and it was not possible to clearly represent a region (urban or rural) due to the sporadic case distribution from all over the country.

In our series, the highest 5-year survival rates were observed for thyroid and breast cancers (87.52% and 73.05%, respectively), and the 5-year survival rates for the five most common malignancies were cancers of the colon-rectum, stomach, lung, esophagus, and liver–with rates of 52.89%, 32.10%, 18.64%, 33.72%, and 19.04%, respectively, showing large differences from the results reported in Shanghai. One possible reason for this difference is that Shanghai is one of the most economically and medically developed areas in China, with high levels of medical care and treatment available at large hospitals. In addition, patients in Shanghai were from urban areas with better access to pre- and post-treatment care and high levels of economic support for diagnosis and treatment. However, it is important to note that overestimation of survival may occur due to low follow-up rates.

An Australian report showed that rural people with cancer had poorer survival after a cancer diagnosis compared to those in major cities, which may be attributed to the fact that older people make up a larger proportion of the population in rural areas (30). In an Indian study, it was also found that population-based cancer survival was lower in rural areas than urban areas (31). Even in high-income countries, worse survival for cancer patients living in rural areas than in urban regions could be observed, such as in France (32) and the USA (33). It was also observed in a Japanese study (34) that there were differences in treatment strategy and survival outcomes among older adult patients with esophageal cancer between metropolitan and non-metropolitan areas due to extensive urban services and medical delivery systems. The relatively poorer survival of patients in rural areas may also be due to the increasing distance from a cancer center, as noted in a Scottish study noted (35).

Increasingly, there are documented cancer care disparities among rural populations, which may worsen, likely due to the difficulty in accessing state-of-the-art cancer prevention, diagnosis, and treatment services in rural areas (36, 37). Available data suggest that there is a need for improved medical care in most areas of China, particularly in rural areas. When comparing cancer survival by country and area, significant differences were observed globally. For example, the 5-year survival rate for breast cancer is close to 90% in the United States and Australia but only 40% in South Africa. The highest 5-year survival rates for gastrointestinal cancers are seen in Southeast Asia, with Korea having the highest survival rates for cancers of the stomach (69%), colon (72%), rectum (71%), Japan for esophageal cancer (36%), and Taiwan for liver cancer (28%). For children diagnosed with leukemia during 2010-2014, five-year survival ranged from 66% in Thailand to 95% in Finland (2). In China (2012-2015) (5), the highest 5-year survival rates were observed for thyroid cancer (84.3%), breast cancer (82.0%), bladder cancer (72.9%), corpus cancer (72.8%), and kidney cancer (69.8%), whereas the lowest were observed for pancreatic cancer (7.2%), liver cancer (12.1%), gallbladder cancer (16.4%), leukemia (25.4%), and bone cancer (26.5%).

According to the CONCORD-3 study worldwide, countries with high cancer survival rates include the United States, Canada, Australia, New Zealand, and Northern European countries (2). For example, PBR data in the United States showed that the 5-year survival rate increased from 49% in 1975-1977 to 68% in 2012-2018 (38). Global studies have also shown higher survival in high-income countries and lower rates in low- and middle-income countries (39). China’s target (6) for the 5-year overall survival for all cancers combined is 43.3% by 2022 and 46.6% by 2030. In this series, the 5-year overall survival from 2007 to 2017 was 40.48%, with 39.35% in 2007-2011 and 41.26% in 2012-2017, indicating a slight improvement during the two periods. Therefore, it is inferred that a target of 43.3% will be achieved by 2022.

In terms of public health, it is not appropriate to directly compare data from the HBR and PBR, but they can still be used as mutual references in practice. It is worth noting that significant regional disparities in the cancer survival rates. The Nantong area is located in Eastern China, with a relatively high level of economic development and health care, yet its survival is similar to the national average and lower than that of urban areas such as Shanghai and developed countries. An early Utha study (40) revealed that the disparities in cancer survival between rural and metropolitan residents may be attributed to differences in the accessibility of screening and treatment. In most underdeveloped countries or regions, the prognosis of patients with cancer is also less favorable due to the level of economic development and the availability of medical resources. A recent report from a population-based cancer registry in Brazil showed that, from 2000 to 2018, pancreatic cancer had the lowest 5-year net survival (5.5%), followed by oesophageal cancer (5.6%), while prostate cancer (92.1%) and thyroid cancer (87.4%) ranked among the highest (41). An Iranian report demonstrated that cancer patients from nine provincial population-based cancer registries during 2014 to 2015 experience relatively poor prognosis compared to those in high-income countries, with 5-year net survival of 12.2%, 13.6%, 14.2% and 19.6% for cancers of pancreas, lung, liver and stomach, respectively (42). These findings indicate that improving cancer survival in developing countries or rural areas remains a challenge and an area with potential for improvement. Therefore, achieving the national goal of 46.6% five-year survival for cancer by 2030 (6) will require a focus on improving cancer survival in the vast rural areas of China.

Several characteristics of cancer survival in the rural area are represented in this document:1) Five-year cancer survival in the rural area was relatively higher in women (51.90%) than in men (30.19%), except for sex-specific cancer sites. This difference is commonly seen in other studies, although the reasons behind sex-specific differences in cancer survival are not well understood (43–45). 2) Young adult patients (20-29 years:69.26%; 30-39 years:60.87%) had higher survival rates than older patients (70-79 years:29.28%; 80-89 years:22.43%). Therefore, aging is a negative prognostic factor for survival outcomes in many cancer sites (46–48). 3) Over the past two period, 5-year cancer survival has improved (39.35% vs. 41.26%), although improvement at some sites was not significant. This implies progress in cancer treatment and improvements in cancer services (20, 49). 4) Five-year survival of cancer patients varies between regions and counties, which may reflect differences in the patterns of major cancers (resulting in disparities in hospitalization proportion). For example, esophageal cancer is relatively common in HA and RG, while liver cancer is more prevalent in QD and HM (25, 27, 50). 5) Additionally, as seen worldwide, cancer survival differ across different cancer sites (1–5, 29–32). For instance, thyroid gland cancer had the highest 5-year survival of 87.52%, while pancreatic cancer had a survival of only 6.37% in this study series. These variations may primarily depend, to a greater or lesser extent, on factors such as disease characteristics, treatment modalities, early detection or screening, and patient demographic factors. Overall cancer survival varies from region to region due to differences in cancer survival and the proportions of different cancer sites. Therefore, when comparing overall survival between countries or within regions and time periods, it is important to consider factors such as age, sex, and the major cancer sites that may affect prognosis.

This study had some limitations. First, the clinical stages of cancers were not analyzed because there were no long-term completed records in the hospital HIS system, and there were no large-scale screening programs in this rural area except for liver cancer screening in some high-risk populations in QD and HM (51, 52). Second, survival by therapy was not available because of the complicated classification of the treatment of malignancies involved in HBR. Third, it is not possible to assess the risk factors for differences in survival for cancers due to the lack of systematic medical history records from the HIS. In addition, the relative survival of the HBR data was not calculated using the PBR data. However, this article provided a realistic observed survival for all patients in a rural area treated at a tertiary cancer hospital, mirroring the survival status after completed follow-up in patients with cancer in a rural area. We believe that this paper provides both a basis for local cancer prognosis evaluation and cancer prevention research and highlights the shortcomings and challenges in cancer treatment in this professional cancer institution.

## 5 Conclusion

This study used mixed methods of repeated active and passive follow-up to maximize the reliability and completeness of the regional follow-up by combining HBR and PBR data within the area, which is crucial for assessing cancer prognosis and conducting cancer survival studies. The results showed that cancer survival was higher in women than in men, and that younger patients had higher survival rates than older patients. Cancer survival was relatively lower in this rural area compared to data from urban areas and high-income countries. However, over the past two periods, cancer survival has improved, although the improvement in some cancer sites was not significant. Enhancing cancer survival in rural areas remains a challenge and an area with potential for improvement.

## Data availability statement

The datasets presented in this article are not readily available because the data of this study can be available upon reasonable request to the corresponding authors. Requests to access the datasets should be directed to chenjg@ntu.edu.cn.

## Ethics statement

The studies involving human participants were reviewed and approved by the Hospital Ethics Committees of Nantong Tumor Hospital/Affiliated Tumor Hospital of Nantong University (NTH-HEC-2020011). The need for informed consent was waived by the Hospital Ethics Committee of Nantong Tumor Hospital / Affiliated Tumor Hospital of Nantong University.

## Author contributions

Conceptualization: G-RW, BC, and J-GC. Data duration: HX, H-ZC, Y-SC, Z-JN, L-YF, A-HZ, P-PX, YQ, and J-GC. Formal analysis: HX, H-ZC, Y-SC, BC, and J-GC. Funding acquisition: None. Investigation: HX, Y-SC, Z-JN, L-YF, A-HZ, P-PX, and YQ. Methodology: HX and J-GC. Project administration: G-RW, BC, and J-GC. Resource: G-RW, BC, and J-GC. Software: Y-SC and J-GC. Supervision: G-RW and J-GC. Validation: HX, H-ZC, Y-SC, BC, and J-GC. Visualization: HX, H-ZC, Y-SC, BC, and J-GC. Writing - original draft preparation: G-RW, HX, and J-GC. Writing - Review and Editing: J-GC.
