# Supplementary material for: Survival of 48866 cancer patients: results from Nantong area, China
Source: Front Oncol. 2023 Aug 11;13:1244545. doi: 10.3389/fonc.2023.1244545 (PMC10455932; doi:10.3389/fonc.2023.1244545)
Supplement: Supplementary file 1 [file Table_1.docx]

S TABLE 1 Distribution of patients with cancer by site, gender and sex ratio.

| **Site** | **ICD-10** | **Sex** | | **Total** | **M/F** |
| --- | --- | --- | --- | --- | --- |
|  |  | **Male** | **Female** |  |  |
| Nasopharynx | C11 | 549 | 217 | 766 | 2.53 |
| Lip, oral & pharynx ^a^ | C00-14 | 571 | 318 | 889 | 1.80 |
| Esophagus | C15 | 5915 | 2803 | 8718 | 2.11 |
| Stomach | C16 | 3389 | 1371 | 4760 | 2.47 |
| Small intestine | C17 | 89 | 63 | 152 | 1.41 |
| Colon | C18 | 719 | 570 | 1289 | 1.26 |
| Rectum & anus | C19-21 | 1099 | 720 | 1819 | 1.53 |
| Liver | C22 | 3000 | 820 | 3820 | 3.66 |
| Gallbladder | C23-24 | 218 | 233 | 451 | 0.94 |
| Pancreas | C25 | 501 | 386 | 887 | 1.30 |
| Other digest. system | 26 | 11 | 6 | 17 | 1.83 |
| Nasal cavity & access. sinuses | C30-31 | 97 | 56 | 153 | 1.73 |
| Larynx | C32 | 213 | 9 | 222 | 23.67 |
| Trachea & lung | C33-34 | 5321 | 2503 | 7824 | 2.13 |
| Thymus, heart, mediast. & pleura | C37-38 | 84 | 67 | 151 | 1.25 |
| Bone & articular cartilage | C40-41 | 51 | 45 | 96 | 1.13 |
| Malignant melanoma | C43 | 117 | 112 | 229 | 1.04 |
| Skin (except C43) | C44 | 233 | 224 | 457 | 1.04 |
| Mesothelium & soft tissue | C45-49 | 83 | 94 | 177 | 0.88 |
| Breast | C50 | 31 | 3260 | 3291 | 0.01 |
| Female genital organs ^b^ | C51-58 | - | 332 | 332 | - |
| Cervix uteri | C53 | - | 4449 | 4449 | - |
| Corpus uteri | C54 | - | 823 | 823 | - |
| Ovary | C56 | - | 1152 | 1152 | - |
| Penis | C60 | 85 | - | 85 | - |
| Prostate | C61 | 478 | - | 478 | - |
| Testis & other male genital organs | C62-63 | 45 | - | 45 | - |
| Urinary tract ^c^ | C64-68 | 242 | 135 | 377 | 1.79 |
| Bladder | C67 | 389 | 96 | 485 | 4.05 |
| Eye & adnexa | C69 | 6 | 4 | 10 | 1.50 |
| Brain & CNS | C70-72 | 176 | 151 | 327 | 1.17 |
| Thyroid gland | C73 | 210 | 519 | 729 | 0.40 |
| Other endocrine glands | C74-75 | 13 | 11 | 24 | 1.18 |
| Unknown, NOS ^d^ | C76-80 | 763 | 812 | 1575 | 0.94 |
| Hodgkin's disease | C81 | 58 | 38 | 96 | 1.53 |
| Non-Hodgkin's lymphoma (NHL) | C82-85 | 782 | 574 | 1356 | 1.36 |
| Multiple myeloma | C88-90 | 48 | 46 | 94 | 1.04 |
| Leukemia | C91-96 | 41 | 29 | 70 | 1.41 |
| Independ. Multiple sites | C97 | 101 | 90 | 191 | 1.12 |
| All sites | C00-97 | 25728 | 23138 | 48866 | 1.11 |

**Notes:** ^a^ Except C11; ^b^ Except C53, C54, C56; ^c^ Except C67; ^d^ Include ill-defined, secondary & unspecified sites. -, not available.

**Abbreviations:** ICD-10, International classification for diseases – 10th version; Brain & CNS: Brain and central nerves system; NOS, Not otherwise specified.
